# Response Surface Methodology for Optimization of Rotating Biological Contactor Combined with External Membrane Filtration for Wastewater Treatment

**DOI:** 10.3390/membranes12030271

**Published:** 2022-02-27

**Authors:** Sharjeel Waqas, Noorfidza Yub Harun, Muhammad Roil Bilad, Taufik Samsuri, Nik Abdul Hadi Md Nordin, Norazanita Shamsuddin, Asep Bayu Dani Nandiyanto, Nurul Huda, Jumardi Roslan

**Affiliations:** 1 Chemical Engineering Department, Universiti Teknologi PETRONAS, Seri Iskandar 32610, Perak, Malaysia; sharjeel_17000606@utp.edu.my (S.W.); noorfidza.yub@utp.edu.my (N.Y.H.); nahadi.sapiaa@utp.edu.my (N.A.H.M.N.); 2 Faculty of Integrated Technologies, Universiti Brunei Darussalam, Gadong BE1410, Brunei; norazanita.shamsudin@ubd.edu.bn; 3 Faculty of Applied Science and Technology, Universitas Pendidikan Mandalika (UNDIKMA), Jl. Pemuda No. 59A, Mataram 83126, Indonesia; taufiksamsuri@ikipmataram.ac.id; 4 Department of Chemistry, Universitas Pendidikan Indonesia, Bandung 40154, Indonesia; nandiyanto@upi.edu; 5 Faculty of Food Science and Nutrition, Universiti Malaysia Sabah, Kota Kinabalu 88400, Sabah, Malaysia; jumardi@ums.edu.my

**Keywords:** attached growth process, biofilm, biological wastewater treatment, response surface methodology, analysis of variances, Box–Behnken design, membrane fouling, rotating biological contactor

## Abstract

A large amount of wastewater is directly discharged into water bodies without treatment, causing surface water contamination. A rotating biological contactor (RBC) is an attached biological wastewater treatment process that offers a low energy footprint. However, its unstable removal efficiency makes it less popular. This study optimized operating parameters in RBC combined with external membrane filtration (RBC-ME), in which the latter acted as a post-treatment step to stabilize the biological performance. Response surface methodology (RSM) was employed to optimize the biological and filtration performance by exploiting three parameters, namely disk rotation, hydraulic retention time (HRT), and sludge retention time (SRT). Results show that the RBC-ME exhibited superior biological treatment capacity and higher effluent quality compared to stand-alone RBC. It attained 87.9 ± 3.2% of chemical oxygen demand, 45.2 ± 0.7% total nitrogen, 97.9 ± 0.1% turbidity, and 98.9 ± 1.1% ammonia removals. The RSM showed a good agreement between the model and the experimental data. The maximum permeability of 144.6 L/m^2^ h bar could be achieved under the optimum parameters of 36.1 rpm disk rotation, 18 h HRT, and 14.9 d SRT. This work demonstrated the effective use of statistical modeling to enhance RBC-ME system performance to obtain a sustainable and energy-efficient condition.

## 1. Introduction

Stringent effluent standards imposed by regulatory authorities highlight the need to develop a sustainable and environmentally-friendly treatment process [1,2]. Because of the increase in the volume and the pollution level of wastewater, the treatment costs also increase. Various treatment methods employed for wastewater treatment are ozonation, photocatalytic oxidation, and biological processes. Ozonation is a chemical water treatment technique based on the infusion of ozone into water. The treatment of water with ozone has a wide range of applications, as it is efficient for disinfection as well as for the degradation of organic and inorganic pollutants. Ozonation is not economical for poor quality (poorly treated) wastewater. Ozone is extremely irritating and possibly toxic, so off-gases from the contactor must be destroyed to prevent worker exposure. The cost of treatment is relatively high, being both capital- and power-intensive. Physical–chemical oxidation is used in the treatment of water for a range of purposes: for disinfection before household or industrial using in order to avoid any danger of bacterial contamination; and for precipitating dissolved compounds (iron, manganese, sulphides). Photocatalytic oxidation is a very powerful air purification technology and has the ability to destroy particles as small as 0.001 microns (nanometer). The technology works by breaking down the pollutants to improve the effluent quality. However, photocatalytic oxidation technology possesses several advantages, including environmental protection, the complete degradation of pollutants, and no secondary pollution. The major limitations are a lack of solar sensitivity and lower efficiency, which hinder widespread application. Biological methods have been widely adapted in wastewater treatment with the advantages of being more cost-efficient, having more minor footprint requirements, higher specific biomass activities, and being sustainable and environmental-friendly [3,4].

Rotating biological contactors (RBC) rely on the rotating submerged disks to grow biofilm that is kept in constant motion for energy-efficient aeration. Longer sludge retention time (SRT) allows the growth of the nitrifying community throughout the biofilm to perform complete nitrification [5]. In RBC, sufficient microbial growth on the rotating disks exploits the benefit of high effluent quality and treatment efficiency. The RBC bioreactor has been used for nitrogen removal with acceptable removal efficiencies. It operates at a high microbial concentration that allows a higher organic loading rate [6]. As in the membrane bioreactor, it can further be extended by incorporating membrane filtration for sludge separation. Recently, various RBC configurations have appeared as promising alternatives to the traditional treatment processes, and they have been applied for the treatment of municipal and industrial wastewaters [7].

Biological wastewater treatment incorporating membrane separation has been the focus of research worldwide [8,9,10]. However, membrane fouling is a significant concern of membrane technology and should be seriously considered [11]. Operational parameter optimization can reduce membrane fouling, improve membrane properties, and tweak hydrodynamics near the membrane surface [12]. Optimization of operational parameters plays a vital role in biological wastewater treatment [13,14,15].

The relationship and optimization of the operational parameters can help increase the bioreactor’s performance efficiency [16]. Hence, the design of experiments (DOE) is an excellent choice to optimize the process parameters. The DOE acquires fewer experiments and is statistically predictable with highly reliable and efficient results. Response surface methodology (RSM) is an excellent example of such an approach in enhancing bioreactors’ performance efficiencies and simultaneously studying various parameters’ behaviors [17]. RSM is an empirical statistical technique that can investigate mathematical modeling to comprehend the mutual relationship of various process parameters on the response variable. The quantitative data generated from experimental design and regression model analysis, as well as operational conditions can result in high-end performance [18]. Some studies have focused on optimizing the operating conditions of the membrane process. Askari et al. [19] studied RSM to examine the effect of process conditions on the NF membrane removal efficiency. Yang et al. [20] applied RSM design and CFD simulation to optimize hydrodynamics for cake layer fouling control. The application of RSM design helps to optimize the multiple parameter operating system at the expense of maximum membrane fouling.

Instigated by the success of the conventional activated sludge process incorporating membrane filtration, an RBC bioreactor combined with membrane filtration (RBC-ME) has been proposed [15]. Membrane integrated RBC employs membrane technology as post-treatment, eliminating the need for a settling tank. The operational parameters, namely SRT, hydraulic retention time (HRT), and disk rotational speed, influence microbial activity and membrane fouling potential [21]. These operational parameters can alter microorganism properties and help in optimizing the system’s performance. Recent studies have depicted that disk rotational speed, HRT, and SRT influence biodegradation and micro-pollutant removal in membrane systems [22,23]. The selection of short HRT results in a high organic loading rate, increasing the viscosity and sludge concentration and increasing the filamentous bacteria growth [13]. This study evaluates the effect of operational parameters on membrane fouling potential without employing any membrane fouling control techniques.

This study utilized RSM modeling and optimization to investigate the relationship between variables by establishing the predicted models. Box–Behnken design (BBD) through RSM was employed to examine the effect of three operational parameters (disk rotational speed, HRT, and SRT) on membrane permeability. This study investigated the relationship between operational parameters (disk rotational speed, HRT, and SRT) and the permeability response parameter and found the process’s optimal condition using RSM. Experiments under variable operational parameters (disk rotation, HRT, and SRT) were conducted. Then, the performance of an RBC combined with an external membrane filtration (RBC-ME) bioreactor was analyzed.

## 2. Materials and Methods

### 2.1. Wastewater Preparation and Bioreactor Acclimatization

The lab-scale RBC-ME bioreactor was fed with synthetic wastewater prepared by blending food leftovers [12]. The use of synthetic wastewater was chosen to maintain the same composition along the testing period and to allow independent investigation on the effect of studied parameters. Table 1 summarizes the influent wastewater concentrations.

### 2.2. Membrane Preparation and Characterization

A flat sheet polysulfone (PSF) membrane was fabricated in-house and used during the experiments. The membrane was fabricated using PSF as polymer, polyethylene glycol (PEG) as an additive, and dimethylacetamide (DMAc) as solvent through the phase inversion technique. PSF, PEG, and DMAc were used at concentrations of 12%, 1%, and 87%, respectively. Our previous study gives a detailed procedure for the manufacturing and characterization of the membrane [24]. The dope solution was kept for 24 h without mixing to release the entrapped air bubbles. Entrapped air bubbles can cause defects in the membrane sheet; hence, an air bubble-free dope solution is necessary. The prepared dope solution was cast through the phase inversion method. The dope solution was cast atop a non-woven support (Novatexx 2471, Freudenberg-Filter, Weinheim, Germany) to avoid shrinkage [25] using a casting knife at 0.22 mm wet thickness, under 30 °C room temperature and 74% air humidity. The cast film was then immediately immersed in the deionized water (acting as the non-solvent) to form a thin porous membrane. The prepared membrane was rinsed with running tap water to remove any traces of solvent and then stored wet in tap water until further use.

The properties of the fabricated PSF membrane were determined using established analytical methods listed in Table 2. Membrane thickness was measured using an electronic digital micrometer screw gauge (Mitutoyo 293-340-30 Digital Micrometer, Mitutoyo America Corporation, Aurora, CO, USA). The pore size was measured using a capillary flow porometer (Porolux, Nazareth, Belgium). The membrane morphology was analyzed, and the microstructure was acquired using scanning electron microscopy (SEM) (Zeiss, Leo 1430 VP, Carl Zeiss, Oberkochen, Germany). The static membrane surface water contact angle was determined using the sessile drop method (mobile surface analyzer, KRUSS, Hamburg, Germany). All the characterization techniques were done in triplicate.

### 2.3. Bioreactor Set-Up

**Figure 1:**
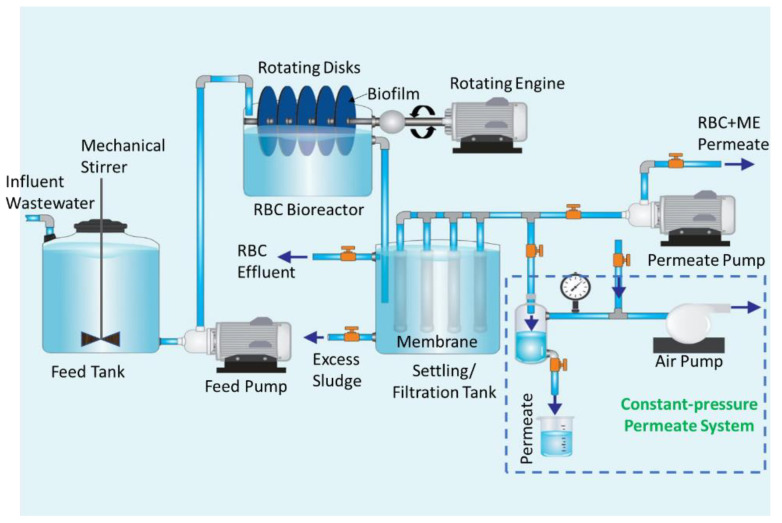
Schematic diagram of RBC-ME configuration.

The RBC-ME bioreactor consisted of a feed wastewater tank, RBC bioreactor, and external membrane vessel constructed in-house, as shown in Figure 1. The feed wastewater tank of capacity 42 L was used as a storage vessel to supply a constant wastewater supply to the RBC bioreactor. The feed tank was facilitated with a mechanical stirrer (stirred at 100 rpm) to maintain a uniform concentration of wastewater throughout the storage vessel. The RBC bioreactor consisted of a 25 × 25 × 30 cm tank fabricated from poly (methyl methacrylate) and had a working volume of 6.5 L. The bioreactor was equipped with a stainless-steel shaft with five rotating disks of 1.8 cm thickness and 18 cm diameter. The shaft was attached to a DC motor that rotates the disks. The poly (methyl methacrylate) disks had a net surface area of 2034 cm^2^ and were placed inside the RBC bioreactor at 40% submergence. Polyurethane sheets (1.22–1.27 g/cm^3^ density) were attached to the disks acting as a substrate for biofilm formation. The flat sheet membrane module was placed after the RBC bioreactor acting as a post-treatment without any means of fouling control.

The RBC-ME system did not include a settling tank (typically a part of a conventional RBC unit), as it was replaced with a membrane filtration tank. Two membrane sheets with an overall effective area of 226 cm^2^ were glued on both sides of a panel frame surface using A–B glue (A–B quick epoxy, HYRO) to form a two-sided plate-and-frame filtration panel, as detailed elsewhere [26]. The membrane filtration tank had a working volume of 5 L, and a single membrane panel was submerged. The permeate was collected from the filtration cell, and its volume was measured regularly.

### 2.4. Bioreactor Operation

The lab-scale RBC-ME bioreactor was inoculated from the activated sludge obtained from the nearly full-scale domestic wastewater treatment plant. The bioreactor was operated for 30 days, divided into two phases. The bioreactor was operated for 15 days during the first phase to acclimatize the biofilm fully. The organic loading rate of the influent wastewater was kept constant at 19 g chemical oxygen demand (COD)/m^2^ d, and biofilm formed at the rotating disks was observed for any changes. After biofilm acclimatization, the membrane panel was placed into the system to study the impact of membrane permeability performance under various parameters. More details on the bioreactor operation can be found in our previous study [24].

The bioreactor’s experimental investigation was done for three operating parameters (disk rotational speed, HRT, and SRT). The disk rotation was set from 30 to 50 rpm, HRT was set from 9 to 15 h, and SRT was set from 5 to 15 days. The disk rotational speed increased with the variable speed DC motor; the flow rate to the bioreactor was increased to increase the HRT, while SRT was varied by wasting a portion of the reactor volume each day. The HRT and SRT are referred to as the RBC bioreactor parameters. The changes in HRT and SRT impact the microbial community’s biological degradation activity. HRT can be adjusted by changing the influent wastewater flowrate to the bioreactor. HRT for the bioreactor was calculated by using Equation (1).

(1) $$HRT=\frac{V_{T}}{Q}$$

where *HRT* is the hydraulic retention time (h), $V_{T}$ total reactor volume (L), and Q volumetric flowrate (L/h).

### 2.5. Analytical Methods

COD, total nitrogen (TN), ammonia, and nitrate were measured using each compound’s specific Hach digestion solution (HACH, Loveland, CO, USA). Each sample was diluted to fall into the range of the digestion vials being used for the study. The values were determined using a Hach DR3900 Spectrophotometer (HACH, Loveland, CO, USA). A Hach 2100Q portable turbidimeter (HACH, Loveland, CO, USA) and Hach HQ411D benchtop PH/MV meter (HACH, Loveland, CO, USA) were used to determine turbidity and pH, respectively [27].

### 2.6. Determination of Filtration Performance

The low pressure for filtration reduces the energy cost, is less susceptible to membrane fouling, and maintains sustainable flux, as reported elsewhere [28]. The membrane permeability (L, L/m^2^ h bar) was calculated by using Equation (2).

(2) $$L=\frac{∆V}{A\;∆t\;∆P}$$

where *V* is the volume of permeance (*L*), *A* membrane area (m^2^), *t* filtration time (h), and ∆*P* transmembrane pressure (bar).

### 2.7. Shear Rate Calculation

Shear stress is the force acting tangential to the surface due to friction and is proportional to the rate of deformation tensor. Shear stress was estimated according to the simulated velocity profile [29]. The effect of shear rate is dominant toward the disk outer sides as a result of the inferior linear velocity compared to that at the disk center, as presented in Equation (3). The disk rotation near the stationary surface stimulates the shear rate, as shown in Equation (4).

(3) $$\gamma =\frac{\nu }{h}$$

(4) ν=rω

where v is the rotating disk linear velocity (m s^−1^), r the distance from the center towards the outer disk edge (m), ω the angular velocity of rotating disk (rad s^−1^), γ the shear rate induced by the rotating disk, and h the gap (m).

### 2.8. Experimental Design by RSM Method

RSM is an aggregation of mathematical and statistical approaches accommodating to examining various operational parameters’ effectiveness. Design-Expert software (DES) version 8.0.6 was applied to evaluate the response of various parameters [30,31]. DES not only analyzes the parameters; it also helps to determine the projected outcomes to compare with the actual values. It can also help predict the optimized values for the independent operational parameters [32,33].

The application of RSM to design optimization reduces the cost of expensive analysis methods and their associated numerical noise. The response variable can be represented graphically (contour plots or three-dimensional space) to help visualize the response surface’s shape. The RSM principle is based on two fundamental concepts: selecting the approximate model, and evaluating the response. The selection of an approximate model is helpful to obtain the optimized solution at the expense of minimum experimentation. The objective of DOE is the selection of the points where the response should be evaluated.

To scrutinize the impact of various operational parameters on the membrane fouling propensity in RBC-ME configuration, a 3-variable with 3-level BBD model was employed, holding 3 central points per block. A 3-level BBD depicts that each numeric factor is varied over 3 levels. Three independent operational parameters are (i) disk rotational speed, (ii) HRT, and (iii) SRT. The BBD consists of three levels for all operating parameters: high level or maximum (referred to as +1), medium level or central (referred as 0), and low level or minimum (referred as −1). The disk rotations were varied from 30 to 50 rpm; HRT was in the range of 12–18 h, while SRT varied from 5 to 15 days. The independent variables and their range utilized are listed in Table 3. The three independent variables are presented as A, B, and C, respectively, for statistical computations. The resulting response of membrane permeability was determined to investigate the influence of variables. The results of response date were analyzed with analysis of variance (ANOVA) if liable terms composed a powerful effect (*p* ≤ 0.05). Model fit and transformation to graphs were evaluated to interpret and evaluate the model prediction results.

## 3. Results and Discussion

### 3.1. Membrane Characterization

The properties of the applied membrane in the external filtration system are summarized in Table 3. The thickness and mean flow pore size were 0.28 ± 0.22 mm and 0.03 µm, respectively. The sizes of the microorganism species were much larger than the mean flow pore size of the membrane combined with the asymmetric nature of the morphology, hence ensuring complete biomass retention at the membrane surface. For an asymmetric phase inverted membrane, the membrane pore size is dictated by the size of the pore mouth [34], which in this context disallowed penetration of any free biomass into the membrane structure. This advantage ensures no carry forward of biomass to the effluent and any suspended matter typically vulnerable in a standard settling system. However, colloidal particles and dissolved nutrients can pass through the membrane pores, unless an additional layer of biofilm grows on the membrane surface, which aid in biodegradation, as often occurs in a membrane bioreactor [35]. It is worth noting that the biological performance is less affected by the membrane properties. The membrane surface water contact angle determines the hydrophilic/hydrophobic nature of the membrane. The membrane surface water contact angle of 61.8 ± 1.0° reveals a hydrophilic membrane. The membrane exhibits a clean water permeability of 817 ± 35 L/(m^2^ h bar).

### 3.2. Biological Performance

Table 4 summarizes the biological performance of the RBC and RBC-ME bioreactor employing the PSF membrane for synthetic wastewater treatment. RBC operation stabilized after approximately 30 d of operation ascribed from the profile of pollutant removals. Table 4 shows a stable biological performance as depicted from steady removal efficiencies. The synthetic domestic wastewater used as the RBC feed contained a high number of organics. Therefore, carbonaceous bacteria undertaking the removal of the organics were expected to dominate the biofilm. The abundant carbonaceous bacteria biodegraded the readily available substrate (organic pollutants). The RBC-ME bioreactor demonstrated excellent biological removal efficiencies in COD, ammonia, TN, and turbidity (Table 4).

The results show significantly higher removal efficiencies for the COD, TN, ammonia, and turbidity of 87.6 ± 2.7, 45.2 ± 2.6, 98.5 ± 0.07, and 97.8 ± 0.2, respectively. The higher efficiency for COD and ammonia can be attributed to carbonaceous and ammonia-oxidizing bacteria [36,37]. An increase in effluent nitrate concentration (1.8 ± 0.2) was due to poor anoxic conditions within the biofilm resulting in a low concentration of nitrate oxidizing bacteria. The results reveal that the nitrogen compounds were partially denitrified, leaving the majority of nitrogen compounds in the form of nitrates. Due to the aerobic conditions and high carbonaceous microbial activity, the bioreactor achieved a low TN removal efficiency.

Although RBC-ME showed improved nitrogen removal efficiency compared to the stand-alone RBC bioreactor, the TN removal and denitrification systems were much lower than the reported studies [38,39]. The low TN removal observed in this research could be due to various factors. The disk rotation was only half-submerged in wastewater and exposed the biofilm to oxygen, generating high aerobic conditions in the bioreactor. Disk rotation also shredded the excessive biofilm layer and exposed the inner layer to oxygen, thus facilitating the growth of carbonaceous bacteria. This study used a single bioreactor for organics and nutrient removal. Hence, oxidation of organic matter, nitrification, and denitrification occurred in the single bioreactor. The competition between aerobic heterotrophs and denitrifiers for COD reduces the TN removal efficiency.

In the attached growth system, the relative position of the biofilm to the surface affects the oxygen concentration the least (anoxic) in the deeper part. The formed biofilm on the substrate media was still too thin to exert the required anoxic condition for denitrification. The RBC-ME bioreactor achieved higher removal efficiency for turbidity due to the membrane separation. The results showed that the turbidity value substantially diminished from 14.6 ± 0.1 NTU to 0.32 ± 0.03 NTU in the RBC-ME attributes to 97.8 ± 0.2% removal efficiencies (Table 4).

Replacement of the secondary settling tank with the membrane separation enabled the system to achieve removal efficiency not too dependent on the sludge settling characteristics [40]. Detached sludge from the biofilm could readily be removed from the bioreactor. Removing suspended biomass flocs resulted in higher nitrogen and phosphorous removal efficiencies [15,41]. The solid–liquid separation process ensured high effluent quality in organics and nutrients. Consequently, the biological performance could be enhanced by increasing the SRT.

Domestic wastewater often contains low TN and ammonia concentrations. The low influent concentrations dampen the nitrifying bacteria quantity compared to carbonaceous bacteria. The disk rotation mechanism maintains aerobic conditions throughout the bioreactor, and anaerobic conditions are only found deep inside the biofilm [42]. The low influent concentration of TN and lack of anaerobic conditions halt the denitrification step. Previous researchers also shed light on the high DO concentration inside the bioreactor, resulting in low TN removal efficiency [43,44].

Various researchers have studied the biological performance of the RBC. Wang et al. [45] operated a non-woven RBC to treat municipal sewage. The results showed that both COD and TN removal rates were above 70% under optimized conditions with a maximum COD and TN removal efficiency of 83.12% and 79.13% obtained at DO 0.2 mg/L and C/N 2.3, respectively. The COD removal rate showed a decreasing trend with an increase in DO and C/N. Pakshirajan et al. [46] studied the removal of a textile dye from wastewater through the RBC bioreactor. The results described that 64% decolorized wastewater was obtained with glucose as a carbon source, while a maximum removal of 83% was obtained with 10 g/L glucose, and a maximum COD removal efficiency of 73% was obtained. The addition of glucose increases the performance. However, the bioreactor required a significant amount (1:1) of glucose, which is a disadvantage. These results indicate that the RBC bioreactor is an excellent choice for domestic and industrial wastewater treatment due to high microbial activity and flexible operating parameters. Furthermore, the RBC-ME is very attractive to treat wastewater in the open-air canal of wastewater distribution networks where installation of the system is possible, and the issue of a large footprint is less important.

### 3.3. Statistical Analysis and Model Development

Table 5 displays the three factor BBD matrix showing the independent variable and response of membrane permeability. The three operating parameters were disk rotation, HRT, and SRT. Based on our earlier published data, they were selected according to their contribution to biodegradation and membrane fouling [36]. The three-level BBD was used to perform the analysis with a total of 15 experimental runs. The permeability values over each set of parametric values were used to establish a statistical model.

### 3.4. RSM Model Optimization

Full fractional three-factor BBD was applied to investigate the effects of three independent parameters to model the steady-state membrane permeability. The results of the BBD model were described using ANOVA (Table 6), in which the steady-state membrane permeability was set as the response [30]. The ANOVA results showed that the relationship between steady-state membrane permeability and the experimental investigations fit well with a quadratic model with *p* values < 0.0001. The coded and actual factors for membrane permeability are shown in Equations (5) and (6).

Steady-state membrane permeability (L/m^2^ h bar) = 137.33 − 9.50 A − 0.1250 B + 5.88 C − 3.00 AB + 2.50 AC + 1.75 BC − 9.29 A^2^ − 1.04 B^2^ − 1.54 C^2^ (5)

Steady-state membrane permeability (L/m^2^ h bar) = −39.16667 + 7.48333 × Disk rotational speed + 6.26389 × HRT−1.34167 × SRT−0.1 × Disk rotational speed × HRT + 0.05 × Disk rotational speed × SRT + 0.116667 × HRT × SRT−0.092917 × Disk rotational speed^2^−0.115741 × HRT^2^−0.061667 × SRT^2^ (6)

Equations (5) and (6) were used to predict the steady-state membrane permeability listed in Table 6. The three operational parameter (disk rotation, HRT, and SRT) values demonstrated good agreement between the predicted and experimental data. The significance and authenticity of the proposed model were stated based on different model constraints, such as *p* values, R^2^, adjusted R^2^, and F values. A *p*-value < 0.0500 implies a model as significant and insignificant if *p* values are higher than 0.0500. R^2^ represents the coefficient of determination used to validate the quality of the proposed model. A value of R^2^ close to 1 is preferable. The proposed model depicted an R^2^ value of 0.9951, which indicated that 99.51% variations of steady-state permeability were explicable across the parameter ranges. The adjusted R^2^ value of 0.9862 was close to R^2^ values, depicting the goodness of quadratic model fitting. The higher R^2^ value confirmed the excellent adaptation of the predicted model to the experimental data.

The quadratic model predicted an F-value of 111.89, implying that it was significant, with only a 0.01% probability of it occurring due to noise. The model *p*-value less than 0.05 reveals the significant model terms. A *p*-value > 0.05 implicates the model term as non-significant. Table 6 shows that model terms A, C, AB, AC, BC, and A^2^ were significant, while B, B^2^, and C^2^ were insignificant, which implies that HRT variation did not significantly vary the system performance. The insignificant impact of HRT was due to the low strength of influent wastewater and high microbial activity. The influent wastewater mainly consisted of COD and lacked nitrogen compounds, as does most domestic wastewater. RBC facilitated high microbial activity due to attached biofilm growth; the substrate was consumed quickly, and the required effluent quality was achieved at lower HRTs. Previous studies also validated the advantage of higher microbial activity and lowered HRT in RBC [15,47].

A non-significant value for lack of fit is required for a well-fit model. ANOVA results showed a 1.06 lack of fit, which implicated a non-significant relation to the pure error. It indicated that the predicted quadratic model could circumnavigate the design space.

**Figure 2:**
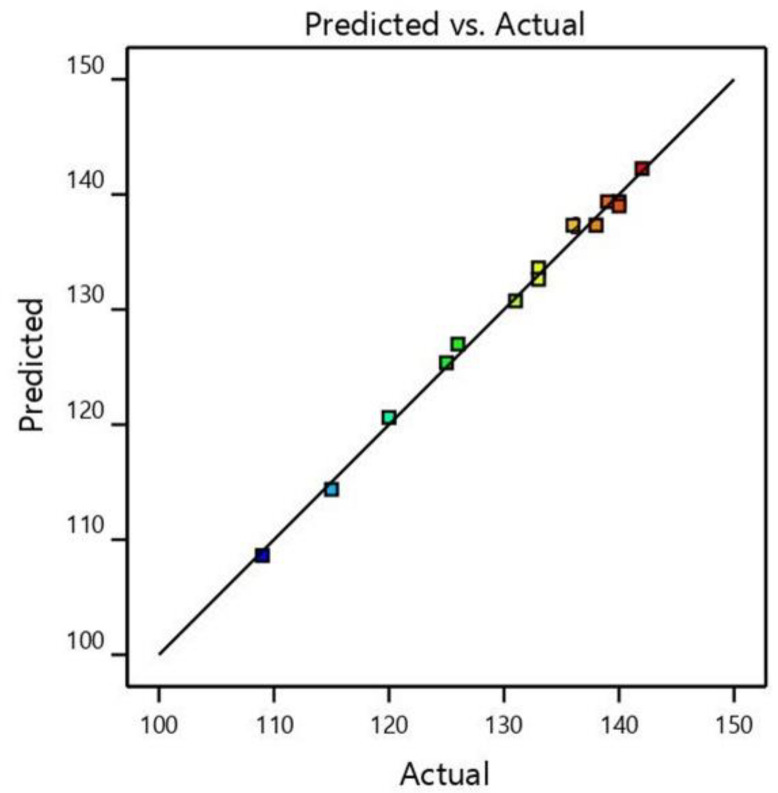
Design-expert plot; predicted vs. actual values plot for steady-state permeability.

A diagnostic plot such as the predicted versus actual values shown in Figure 2 supports adjudicating the model satisfactoriness visually. Figure 2 indicated a satisfactory agreement between actual data and those obtained from the models. A model typically can be considered reproducible if its coefficient of variance (CV) is less than 10%. The quadratic model represents a CV value of 0.8978%, indicating good reproducibility of the model (Table 6).

### 3.5. Process Analysis

**Figure 3:**
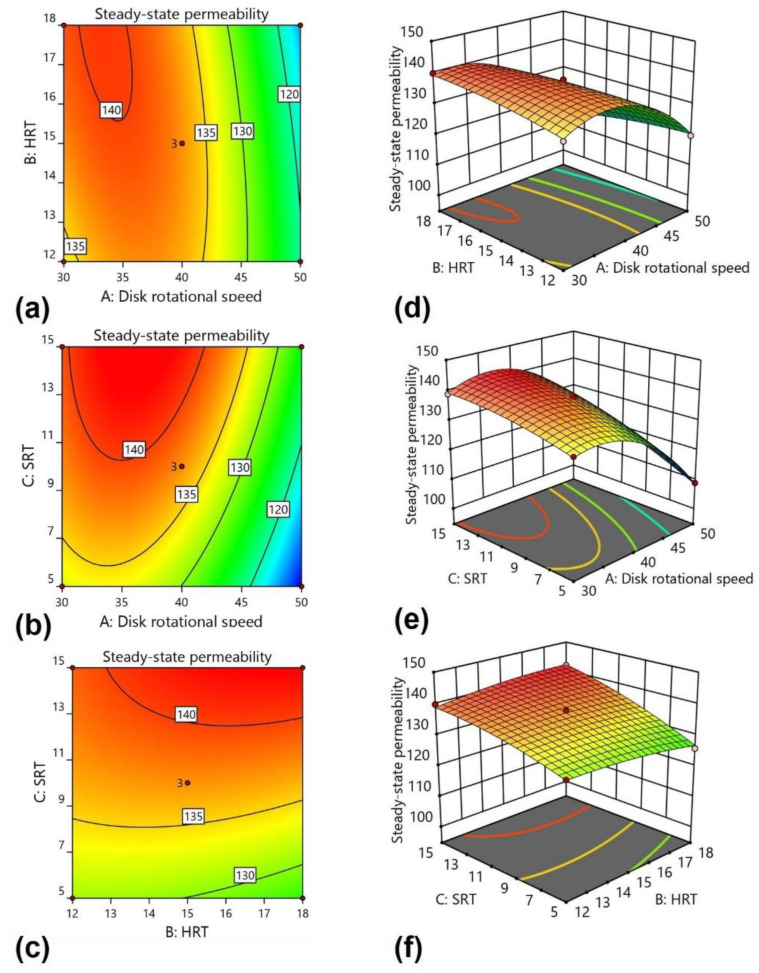
Effect of 2-D contour of (**a**) disk rotational speed and HRT, (**b**) disk rotational speed and SRT, (**c**) HRT, and SRT, and 3-D response surface plots of (**d**) disk rotational speed and HRT, (**e**) disk rotational speed and SRT, and (**f**) HRT and SRT.

Operational parameters affect membrane permeability, which can exist in neutral, positive, or negative configurations. Those parameters can also significantly affect biological performance and effluent quality. Therefore, knowledge of the influence of these parameters on the steady-state membrane permeability can help optimize the whole process (biodegradation and permeability). The dependency of the operational parameters on the membrane permeability is presented in Figure 3 in the form of 2-D contour and 3-D response surface plots. Response surface diagrams illustrate regression equation models utilized to fathom the optimum status of multiple parameters, explain interactions between factors, and ultimately enhance the efficiency of the process. The plots were approximately symmetrical with circular contours. Response surface plots indicated the relative importance of three operating parameters. All response plots demonstrated clear peaks, indicating the optimized parameter (disk rotational speed, HRT, and SRT) values for the maximum permeability in the design space.

The 2-D contour and 3-D response surface plots in Figure 3a,d revealed that the interactions between the disk rotation and HRT significantly affected the permeability. A lower disk rotation and higher HRT resulted in higher permeability. The decline in permeability can be described by the enhanced shear rate produced by higher disk rotational speed—the shear rate generated by disk rotation results in the shredding of biofilm flocs. The biofilm flocs suspended and blocked the membrane pores [48,49]. The permeability decreased as the disk rotational speed increased beyond 40 rpm—the shear rate increased with the increase in disk rotational speed. The shear rate values of 11.3, 15.1, and 18.7 s^−1^ were achieved at 30, 40, and 50 rpm disk rotational speeds, respectively. Higher shear rate values result in a more efficient distribution of dissolved oxygen throughout the bioreactor. However, microorganisms attached to the disk can detach with a higher shear rate and leave suspended floc that does not settle easily. However, a less significant effect was found for the HRT because of the higher organics ratio and lower nitrogen concentration [50]. All the organics were readily degraded and did not require longer HRT, resulting in good performance. Removal efficiencies decreased away from these points, which means that any set of variables beyond this point would decline in permeability.

Figure 3b,e show the 2-D contour and 3-D response surface plots between disk rotation and SRT, depicting that higher disk rotational speed from 30 to 40 rpm at SRT values from 10 to 15 days led to higher permeability, while beyond 40 rpm, the permeability gradually decreased. Shorter SRT resulted in poor settling of sludge, which increased the filtration resistance by blocking the membrane pores [51]. Higher SRT facilitated retention of the microbial community for a longer time in the bioreactor and thus lowered sludge production.

Figure 3c,f show the interaction between HRT and SRT. The results revealed that higher HRT and SRT increased the membrane permeability and dampened the fouling, as reported elsewhere [52]. With the increase of both HRT and SRT from 12 h to 18 h and 5 d to 15 d, respectively, an increase in permeability was observed. Indeed, the membrane permeability further increased at higher HRT and SRT. HRT seemed to affect the permeability slightly. However, high SRT resulted in efficient sludge settling and, subsequently, higher membrane permeability [41]. Overall, high values of both HRT and SRT favored higher permeability.

Results indicate that the membrane fouling was significantly dependent on the operating parameters. Optimal membrane permeability was found at low HRT, moderate disk rotation, and longer SRT. The feed contained a higher carbon compound concentration, which resulted in sufficient carbonaceous microbial community growth. Higher microbial community growth facilitated the decomposition of the substrate at lower HRT. At longer SRT, higher extracellular polymeric substances increased flocculation of particulate and particle sizes, further mitigating membrane fouling [53]. The results agree with the previous research that the outcomes of longer SRT were higher degradation performance for the municipal and industrial wastewater, recalcitrant pharmaceuticals, and micro-pollutants. In principle, the longer SRT is ideal for complete nitrification and high strength wastewater treatment.

### 3.6. Process Optimization

Process optimization of the operational parameters was performed by the mathematical method. The optimal condition of the three process variables for maximum permeability was examined based on the desirability function. Parameter optimization was performed to determine the highest point, which amplified the significant function, i.e., permeability.

Table 7 shows that the optimal conditions for maximum permeability (144.6 L/m^2^ h bar) were as follows: a disk rotation of 36.1 rpm, an HRT of 18 h, and an SRT of 14.9 d. The excellent agreement between predicted and experimental results confirmed model validation to simulate the steady-state permeability.

Two more experiments were performed using the optimum parametric values to confirm the achieved results from the experiments and model. Table 8 shows the experimental and model predictive values for the optimum condition. The steady-state permeability responses of both the experimental and model values were in close agreement.

## 4. Conclusions

This research applied RSM to optimize the filtration performance of an RBC coupled with external membrane filtration. The RBC-ME bioreactor was a successful biological treatment process to achieve a high biological removal efficiency. The RBC exhibited 87.6 ± 2.7% of COD, 45.2 ± 2.6% TN, 98.5 ± 0.07% ammonia, and 97.8 ± 0.2% turbidity removal efficiencies. The RSM results demonstrated the effects of the operating parameters and their interactive effects on permeability as the response. At higher disk rotations (>35 rpm), the permeability decreased due to the higher shear rate and shredding of biofilm flocs. At higher HRT and SRT, higher permeabilities were obtained. By applying RSM, the optimum region for the bioreactor operating conditions was located. The optimum conditions reached a permeability of 144.6 L/m^2^ h at a disk rotation of 36.1 rpm, 18 h HRT, and 14.9 d SRT. The results demonstrated good agreement amongst experimental and model predictions. It is evident that RSM is an efficient statistical optimization approach that can help to distinguish between the most important operational parameters at the cost of minimum time and effort. Developing the membrane-integrated RBC system can significantly enhance the effluent quality to satisfy stringent regulations. It can serve as a promising alternative for decentralized application to develop a sustainable environment.

## Figures and Tables

**Table 1:** Influent characteristics for the RBC-ME bioreactor employing a polysulfone membrane in the post-treatment.

	Influent
COD (mg/L)	282.8 ± 8.3
TN (mg/L)	2.5 ± 0.02
Ammonia (mg/L)	0.64 ± 0.07
Nitrate (mg/L)	0.54 ± 0.02
Turbidity (NTU)	14.6 ± 0.1
pH	6.25 ± 0.03

RBC-ME: rotating biological contactor-membrane external, COD: chemical oxygen demand, TN: total nitrogen.

**Table 2:** Summary of membrane properties used in RBC-ME configuration.

Properties (Unit)	Values
Materials	Polysulfone
Thickness (mm)	0.28 ± 0.22
Mean flow pore size (µm)	0.03 µm
Surface contact angle (°)	61.8 ± 1.0
Cross-section morphology	Asymmetric
Clean water permeability (L/(m^2^ h bar)	817 ± 35

**Table 3:** Independent variables and levels used in Box–Behnken design.

Levels	Independent Variable	Unit	Low Level (−1)	Medium Level (0)	High Level (+1)
1	Disk rotational speed	rpm	30	40	50
2	HRT	h	12	15	18
3	SRT	d	5	10	15

**Table 4:** Effluent characteristics for the RBC-ME bioreactor employing the PSF membrane in the post-treatment.

	RBC Effluent	RBC % Removal Efficiency	RBC-ME Effluent	RBC-ME % Removal Efficiency
COD (mg/L)	78.2 ± 7.5	72.4 ± 2.5	35 ± 7.5	87.6 ± 2.7
TN (mg/L)	1.54 ± 0.05	38.3 ± 1.9	1.37 ± 0.06	45.2 ± 2.6
Ammonia (mg/L)	0.03 ± 0.01	95.6 ± 0.8	0.01 ± 0.01	98.5 ± 0.07
Nitrate (mg/L)	1.9 ± 0.3	--	1.8 ± 0.2	--
Turbidity (NTU)	3.3 ± 0.3	78.9 ± 0.3	0.32 ± 0.03	97.8 ± 0.2
pH	6.82 ± 0.03	--	6.95 ± 0.11	--

RBC-ME: rotating biological contactor-membrane external, COD: chemical oxygen demand, TN: total nitrogen.

**Table 5:** Box–Behnken design matrix for independent variable and response of membrane permeability at three-factor levels.

	Independent Variables			Permeability (L/m^2^ h Bar)	
Run	(A) Disk Rotational Speed (rpm)	(B) HRT (h)	(C) SRT (d)	Actual Value	Predicted Value
1	40	15	10	138	137.33
2	30	12	10	133	133.63
3	30	15	15	139	139.38
4	50	12	10	120	120.63
5	30	15	5	133	132.63
6	40	15	10	136	137.33
7	40	12	5	131	130.75
8	50	18	10	115	114.38
9	30	18	10	140	139.38
10	50	15	5	109	108.63
11	40	18	15	142	142.25
12	40	15	10	138	137.33
13	50	15	15	125	125.28
14	40	18	5	126	127.00
15	40	12	15	140	139.00

**Table 6:** ANOVA results of the coefficient of the quadratic model for steady-state membrane permeability.

Source	Sum of Squares	df	Mean Square	F Value	*p*-Value Prob > F	
Model	1393.08	9	154.79	111.89	<0.0001	Significant
A-Disk rotational speed	722.00	1	722.00	521.93	<0.0001	Significant
B-HRT	0.1250	1	0.1250	0.0904	0.7758	Not significant
C-SRT	276.13	1	276.13	199.61	<0.0001	Significant
AB	36.00	1	36.00	26.02	0.0038	Significant
AC	25.00	1	25.00	18.07	0.0081	Significant
BC	12.25	1	12.25	8.86	0.0309	Significant
A^2^	318.78	1	318.78	230.44	<0.0001	Significant
B^2^	4.01	1	4.01	2.90	0.1495	Not significant
C^2^	8.78	1	8.78	6.34	0.0533	Not significant
Residual	6.92	5	1.38			
Lack of Fit	4.25	3	1.42	1.06	0.5183	Not significant
Pure Error	2.67	2	1.33			
Cor Total	1400.00	14				
Other statistical parameters						
R^2^	Adjusted R^2^	S.D.	A.P.	C.V. (%)		
0.9951	0.9862	1.18	35.0142	0.8978		

**Table 7:** Optimized operational parameter values at maximum steady-state membrane permeability.

		Steady-State Permeability (L/m^2^ h Bar)			
Variables	Optimum Values	Predictive	Experimental	Error (%)	Standard Deviation
Disk rotational speed	36.1 rpm	144.6	144.5	0.44	1.18
HRT	18.0 h				
SRT	14.9 d				

**Table 8:** Steady-state membrane permeability values for the model and experiment.

	Steady-State Permeability (L/m^2^ h Bar)			
Run	Predictive	Experimental	Error (%)	Standard Deviation
1	143.5	143.00	0.35	0.26
2	137.3	137	0.18	0.13

## Data Availability

Not applicable.
